# Comparison of intra-operative outcomes following internal fixation with trochanteric stabilisation plate or intramedullary nail in intertrochanteric fractures

**DOI:** 10.1007/s00590-023-03779-5

**Published:** 2023-11-26

**Authors:** Rahul Geetala, Edward Wakefield, Florence Bradshaw, James Zhang, Matija Krkovic

**Affiliations:** 1 Clinical School of Medicine, University of Cambridge, Addenbrooke’s Hospital, Hills Rd, Cambridge, CB2 0SP UK; 2 Trauma and Orthopaedics Department, Cambridge University Hospitals, Addenbrooke’s Hospital, Hills Rd, Cambridge, CB2 0QQ UK

**Keywords:** Intertrochanteric fracture, Internal fixation, Trochanteric stabilisation plate, Intramedullary nail

## Abstract

**Purpose:**

Intertrochanteric fractures can be classified as stable (AO-OTA 31-A1) or unstable (AO-OTA 31-A2/3). For A3 fractures there is no recommended treatment, often fixed with either an intramedullary nail (IMN) or a dynamic hip screw and trochanteric stabilisation plate (DHS/TSP). This study retrospectively reviews peri-operative outcomes of patients treated with either fixator.

**Methods:**

Pre-operative demographics, operative information and patient outcome data from 213 patients who suffered intertrochanteric fractures and were treated with either DHS/TSP or IMN at a major trauma centre from 01/2015 to 01/2022 was collected. Unpaired *T* tests were performed to assess levels of significance between peri-operative outcomes.

**Results:**

The mean age for DHS/TSP-treated patients was 2.63 years greater than IMN-treated (*P* = 0.039). There were no other significant differences in pre-operative characteristics. We found a significantly shorter mean operative time in the DHS/TSP group (88.05 min, 95% CI: 82.1–94.0) compared to IMN counterparts (100.8 min, 95% CI: 92.7–109.0, *P* = 0.012), but no statistically significant difference in length of hospital stay or patient mortality, blood transfusion, re-operation or complication rates. When A3 fractures were analysed, a statistically significant greater proportion of IMN patients required blood transfusions (DHS/TSP: 35.90%, IMN: 65.00%, *P* = 0.0093). All other factors were found to have no significant differences.

**Conclusion:**

This study provides evidence to support the national guidelines regarding A1 fractures and suggests that DHS/TSP is a valid alternative to the IMN in A3 intertrochanteric fractures, with reduced blood loss. With the flexibility to add the TSP intraoperatively to prevent femoral head medialisation, and cheaper implant costs, the DHS/TSP may become the preferred method of internal fixation.

## Introduction

Intertrochanteric fractures are one of the most common fractures [1], with more complex fractures remaining a challenge to treat and having high re-operation rates [2]. Current NICE guidelines [1] state that the Dynamic Hip Screw (DHS) should be used for fixation of stable intertrochanteric fractures, whilst for unstable A3 intertrochanteric fractures there is no preference between an intramedullary nail (IMN) or a DHS. The DHS relies on having an intact lateral femoral component to adequately stabilise and fix the fracture, preventing further complications such as excessive medialisation of the shaft of the femur [3] associated with worse pain, mobility and an increased risk of operation revision. One way to stabilise these fractures is to maintain the integrity of the lateral femoral wall and offer a secondary point of fixation, which can be achieved through the addition of the Trochanteric Stabilisation Plate (TSP). The TSP prevents excessive medialisation through buttressing of the lateral wall, which prevents secondary displacement and helps fix gluteus medius. Moreover, by providing a secondary point of fixation, the TSP prevents rotation of the proximal femoral component during normal hip movements [4], decreasing the risk of fracture non-union and malunion that can be seen when these fractures are treated with a DHS only. Studies show that the addition of even a custom-made TSP can prevent femur medialisation and collapse [5]. In the context of AO/OTA 31-A2 fractures, the DHS/TSP has been shown to decrease the re-operation rate 13-fold when compared to those treated with DHS only, and reduce the rate of lateral wall fractures [6].

Intramedullary implants provide internal fracture compression and fixation. These implants reduce the moment forces during hip rotation in intertrochanteric fractures and provide more efficient load transfer [7]. Comparisons of the DHS to IMN in unstable intertrochanteric fractures illustrate this, as patients treated with the latter have reduced incidence of fracture non-union, time to weight-bearing and length of operation [8]. However, favourable results for DHS/TSP have been obtained in comparisons with IMN in AO/OTA 31-A3 fractures, with shorter mean operation times for DHS/TSP-treated patients and a significantly diminished post-operative haemoglobin decrease in this group. Therefore, from a surgical perspective, the DHS/TSP seems favourable in these complex, unstable fractures [9]. However, results in German populations contradict this, without reporting any functional difference between these two groups [10].

Therefore, we conducted this retrospective study to investigate patient outcomes using original data from the National neck of femur fracture database and our centre’s internal EPIC™ database, providing further data and evidence to guide the decision on the choice of implant in intertrochanteric fracture fixation.

## Aim

This paper aims to compare short-term patient outcomes following DHS/TSP or IMN fixation of intertrochanteric fractures.

## Methods

A retrospective study was conducted, using the cohort of patients who were diagnosed as having a neck of femur (NOF) fracture at a major trauma centre in the East of England from January 2015 to January 2022, from the national NOF database. From here, these records were compared against patients who received the TSP and long (82 long- and 8 short-stem) IMN prosthesis by searching within the centre’s internal EPIC™ database (PRN 10293). We then selected patients coded as having intertrochanteric fractures and data required to assess patient outcomes was acquired through EPIC™ by a senior author. We imposed a minimum age of 60 years for patients, to exclude fractures in nonelderly patients. Patients were then investigated for the type of fracture with a senior author classifying via AO/OTA classification based on plain radiographs of the hip and the operation notes.

Pre-operative data collected included patient age, gender, side of fracture, fracture classification, ASA grade, pre-operative AMTS, pre-operative 4AT, BMI, pre-fracture residence, pre-fracture mobility and time to surgery. Fractures were classified into stable and unstable as per the AO-OTA classification which classify AO-OTA 31-A2&A3 as unstable. Patients were operated using the standard techniques for both implants under spinal or general anaesthesia. In some cases, a peripheral nerve block was also added intraoperatively. Patients received immediate and regular physiotherapy with the aim for same-day weight-bearing. Weight-bearing was encouraged for as much as the patient could tolerate. Patients were seen by Orthogeriatrics.

Post-operative data collected were grade of surgeon and anaesthetist, anaesthetic used, length of operation, Haemoglobin loss (difference between pre-operative and 24 h post-operation levels), change in Haematocrit (difference between pre-operative and 24 h post-operation levels), length of stay, complications, re-operation rate, number of patients who required a blood transfusion in first 5 days post-operation, discharge destination, and mortality at 30-, 120- and 365-days post-operation. Patients were reviewed by a specialist nurse following the procedure. Unpaired *T* tests was performed to assess levels of significance between peri-operative outcomes. *P* < 0.05 was defined as statistically significant.

## Results

A total of 2697 patients presented with a neck of femur fracture to our centre—of which 947 suffered an extracapsular, intertrochanteric fracture. 203 were treated with either a DHS/TSP (113) or an IMN (90, 82 long- and 8 short-stem) and met our minimum age criteria. The difference between the mean age of both groups was found to be statistically significant, with the DHS/TSP-treated patients on average 2.63 years older than IMN-treated. All other pre-operative data were found not to have statistically significant differences (Table 1). Post-operative measures and outcomes are found in Table 2 (all fractures) and Table 3 (A3 fractures only). Key operative finding includes statistically significant differences in the mean operative times between the two groups, 14.8 min shorter for the DHS/TSP group. When data for A3 fractures only was examined, IMN patients were significantly more likely to require blood transfusions than DHS/TSP (65.00% compared to 36.59%) (Table 3).

**Table 1:** Pre-operative characteristics and social demographics of patients treated with DHS/TSP and IMN

Descriptors	DHS/TSP			IMN			*P* value
	Total *N* = 113 (%)		95% confidence interval	Total *N* = 90 (%)		95% confidence interval	
Male, n	30	26.55%		29	32.22%		
Female, n	83	73.45%		61	67.78%		
Mean age, years	84.99		84.99 ± 1.57 (83.4 to 86.6)	82.36		82.36 ± 1.95 (80.4 to 84.3)	0.039*
Right-sided fracture, n	60	53.10%		41	45.56%		
Left-sided fracture, n	53	46.90%		49	54.44%		
*Intertrochanteric Fracture Grade (AO/OTA)*							
A1	12	10.62%		13	14.44%		
A2	63	55.75%		37	41.11%		
A3	38	33.63%		40	44.44%		
Mean ASA	2.97		2.97 ± 0.119 (2.85 to 3.09)	3		3.00 ± 0.55 (2.85 to 3.15)	0.78
Mean pre-operative AMTS	6.92	No record for 4 patients	6.92 ± 0.672 (6.25 to 7.59)	7.24	No record for 1 patient	7.24 ± 0.73 (6.5 to 7.97)	0.54
Mean pre-operative 4AT	2.17	No record for 18 patients	2.17 ± 0.548 (1.62 to 2.72)	1.60	No record for 17 patients	1.60 ± 0.54 (1.06 to 2.15)	0.20
Mean Body Mass Index	24.13	No record for 2 patients	24.12 ± 1.05 (23.1 to 25.2)	23.90		23.90 ± 1.31 (22.6 to 25.2)	0.79
*Residence*							
Own home/sheltered housing	91	80.53%		77	85.56%		
Nursing home	9	7.96%		3	3.33%		
Residential care	13	11.50%		9	10.00%		
Rehabilitation Unit	0	0.00%		1	1.11%		
*Pre-fracture Mobility*							
Freely mobile without aids	37	32.74%		31	34.44%		
Mobile outdoors with one aid	38	33.63%		22	24.44%		
Mobile outdoors with two aids or frame	23	20.35%		22	24.44%		
Some indoor mobility but never goes outside without help	15	13.27%		15	16.67%		
No functional mobility	0	0.00%		0	0.00%		
*Time to treatment*							
Time to surgery (hours)	26.94		26.94 ± 2.94 (24 to 29.9)	27.85		27.85 ± 3.04 (24.8 to 30.9)	0.68
< 36 h	88	77.88%		73	81.11%		
> 36 h	25	22.12%		17	18.89%		

*Denotes *P *<0.05

**Table 2:** Post-operative measures and outcomes across all fracture subtypes

Patient Outcomes	DHS/TSP *N* = 113	95% confidence interval	IMN *N* = 90	95% confidence interval	*P* value
Mean operation time (minutes)	86.00	86 ± 5.71 (80.3 to 91.7)	100.80	100.8 ± 8.1 (92.7 to 109)	0.0033*
Proportion of operations performed by orthopaedic consultants (%)	88.50		94.44		0.14
Proportion of operations with anaesthetic consultants (%)	65.49		57.78		0.35
Weight-beared within 24 h of surgery (%)	85.84		91.11		0.24
Mean Hb Loss (g/dL)	26.64	26.64 ± 2.87 (23.8 to 29.5)	23.82	23.82 ± 3.28 (20.5 to 27.1)	0.21
Proportion requiring blood transfusion (%)	52.21		58.89		0.34
Proportion requiring blood transfusion > 2 Units (%)	4.42		4.44		0.99
Mean total stay (days)	15.87	15.87 ± 1.79 (14.1 to 17.7)	17.27	17.27 ± 3.07 (14.2 to 20.3)	0.42
Re-operation rate (%)	7.08		5.56		0.66
Discharged home (%)	34.51		47.78		0.056
Discharged to rehabilitation Unit (%)	34.51		24.44		0.12
Discharged to nursing care (%)	13.27		10.00		0.48
Discharged to acute hospital (%)	0		4.44		0.021*
Died at hospital (%)	7.08		6.67		0.91
Mortality Rate at 30 days post-operation (%)	7.08		8.89		0.67
Mortality Rate at 120 days post-operation (%)	18.58		20.00		0.80
Mortality Rate at 365 days post-operation (%)	30.97		28.89		0.75

*Denotes *P* < 0.05

**Table 3:** Post-operative measures and outcomes with AO-OTA 31-A3 fractures

Patient Outcomes	DHS/TSP*N* = 39	95% confidence interval	IMN *N* = 40	95% confidence interval	*P* value
Mean operation time (minutes)	94.10	94.1 ± 11.3 (82.8 to 105)	108.00	108 ± 11.1 (96.9 to 119.1	0.074
Proportion of operations performed by orthopaedic consultants (%)	84.62		92.50		0.28
Proportion of operations with anaesthetic consultants (%)	64.10		57.50		0.46
Weight-beared within 24 h of surgery (%)	87.18		92.50		0.44
Mean Hb Loss (g/dL)	25.76	25.76 ± 4.81 (21 to 30.6)	21.63	21.63 ± 5.05 (16.58 to 26.68)	0.25
Proportion requiring blood transfusion (%)	35.90		65.00		0.0093*
Proportion requiring blood transfusion > 2 Units (%)	2.56		5.00		0.58
Mean total stay (days)	15.55	15.55 ± 2.93 (12.6 to 18.5)	16.93	16.93 ± 4.57 (12.36 to 21.5)	0.60
Re-operation rate (%)	10.26		10.00		0.97
Discharged home (%)	38.46		50.00		0.31
Discharged to Rehabilitation Unit (%)	28.21		22.50		0.65
Discharged to Nursing Care (%)	15.38		10.00		0.53
Discharged to acute hospital (%)	0		3		0.076
Died at hospital (%)	5.13		5.00		0.98
Mortality Rate at 30 days post-operation (%)	7.69		5.00		0.67
Mortality Rate at 120 days post-operation (%)	10.26		10.00		0.97
Mortality Rate at 365 days post-operation (%)	20.51		20.00		0.96

*Denotes *P* < 0.05

Eight patients in the DHS/TSP group required re-operation with four of these being due to infection, in these cases, the patients required several washouts with only one of these patients having the metalwork removed. In the IMN group, there were five cases of re-operation, two of which were due to infection. Interestingly, one of the patients in the IMN group required an amputation on the same limb three and a half years after the initial operation; however, this was unrelated to the initial hip fracture (due to acute limb ischaemia). The difference in mortality, complication and re-operation rates was found to be statistically non-significant between the two groups across all fractures and for the A3 fractures subgroup (Tables 2 and 3).

## Discussion

This paper aimed to investigate patient outcomes in both stable and complex intertrochanteric fractures. Compromised integrity of the lateral wall increases the risk of re-operation, as this area is thought to reduce the chance for collapse and rotation [11]. In these cases, a buttress is required, either through the form of the TSP or the IMN. These structures improve fracture stability [12] and have been shown to improve fixation rates and reduce non-union and re-operation rates [4, 8, 13]. These comparable results suggest that the TSP may also be used in place of the IMN, as both devices allow patients to weight-bear shortly after the operation.

### Length of operation

We found that the operating time for DHS/TSP group was on average 14.8 min shorter than those treated with the IMN, a statistically significant difference. This difference might be due to familiarity of this operating technique, as this is one of the first procedures taught during core surgical training. However, in terms of surgeon skills, we found there were no significant differences in the number of operations performed by consultants for both implants, suggesting that overall, the average familiarity of the surgeon with the operation was the same. When data for only A3 fractures are examined (Table 3), we can see a trend of 13.9 min shorter operations for DHS/TSP, but this difference is non-significant (*P* = 0.074). This suggests that even in complex fractures, fixation with DHS/TSP can be a more efficient procedure than fixation with IMN. When comparing this to results in the literature, we found that there were mixed results with some studies obtaining similar results [9], and others suggesting that IMN had shorter operating times [10, 14, 15], or no significant difference in operating time [16]. Whilst a small fraction of our patients received the short-stem IMN, their average operation time was marginally longer than the DHS/TSP procedure (89 vs 86 min); however, the cohort size is too small (8 patients) to make adequate assessment of the length of the short-stem IMN operation. With our larger sample size than one of the contradicting studies [14], we believe that fixation with the DHS/TSP is a shorter procedure and thus has benefits in terms of freeing up theatre spaces.

### Blood loss

With the invasive nature of these operations, there is often significant blood loss in these patients, requiring blood transfusion. We chose not to use an estimate of blood volume and loss, using the change in Haemoglobin or Haematocrit as a function of blood loss. When comparing the two groups, there was no significant difference in the change in Haemoglobin or Haematocrit. This was also echoed in our comparison between A3 fractures only. Whilst there was no significant difference in the proportion of patients in each group that required a blood transfusion across all fractures, we found a significantly greater proportion (28.41% more) of IMN-treated patients required blood transfusion in A3 fractures compared to DHS/TSP. Although IMN is typically performed as a closed reduction, this might be due to greater removal of trabecular bone in this procedure, causing a greater decrease in Hb concentration, as seen in Fu and Colleague’s study [9]. Our data suggest that treatment with the DHS/TSP leads to less blood loss in unstable fractures compared to the IMN, a significant improvement in a key patient outcome. Results in the literature have also been mixed with some studies suggesting greater blood loss and transfusion requirement in DHS/TSP-treated fractures [15], and other studies suggesting no difference [14, 16]. It is possible that using the shorter length IMN would have reduced blood loss, but 3/8 patients who received short-stem IMN required blood transfusion postoperatively. Overall, our study illustrates that the DHS/TSP performs just as well as the long IMN but may have advantages in the form of shorter operating times and reduced blood loss.

### Re-operation rates

Our study also showed that there was no significant difference in re-operation rates between the two groups. Results in the literature are mixed with studies indicating no long-term differences in unstable fractures [17] or that the DHS/TSP is superior to IMN due to lower incidences of femoral shaft fractures both during and post-operation [18]. This is contradicted by a meta-analysis and retrospective study indicating the IMN had statistically significant lower revision rates [15, 16]. One further study into patients with concomitant ipsilateral femoral neck and intertrochanteric fractures demonstrated statistically significant lower incidences of both fracture non-union and excessive sliding (lag-screw sliding > 15 mm) in the IMN group [19]. Furthermore, biomechanical studies have shown that the TSP/DHS provides similar stability and resistance to femoral shaft medialisation as the IMN [20, 21]. With conflicting results in the literature and our study, a large-scale study investigating this over a long follow-up period would be beneficial to further elucidate any differences, with one RCT reporting no long-term differences in post-operative pain at 3- and 12 months post-operation [17].

Moreover, the TSP is used in fractures of patients when there was failure of using the nail or the DHS on its own, further highlighting the importance of the TSP for stability but also that it will be typically used in more severe cases or in patients where the lateral wall may fracture due to osteoporosis. This can be seen in studies comparing IMN with the DHS, where a much higher percentage of A3 fractures are treated with DHS/TSP than A2 and even more so than A1 fractures [22]. The international success of treatment with DHS/TSP seen in India [23] and South Korea [24] illustrates the utility of this prosthesis to treat intertrochanteric fractures.

### Cost

Although patient care should not be compromised for cost-cutting measures, when there are no significant differences between the two constructs, the more economical implant will be chosen. The reduced price of this DHS/TSP construct, threefold less for small nails and fivefold less for long nails [1] means that overall for these more stable intertrochanteric fractures, there is no reproducible sustained evidence to support using an IMN over a DHS. Despite the longer period before patient weight-bearing, the slightly shorter hospital stays for DHS/TSP are also very appealing as it further reduces the cost of patient stay—however, in our study there were only non-significant trends towards earlier patient post-operative weight-bearing. A small prospective RCT [25] indicated no difference in patient outcomes in unstable fractures with the DHS/TSP over the IMN, but the evidence otherwise illustrates the functional benefits of the TSP.

Overall, the DHS/TSP performs just as well as the IMN but may have advantages in the form of shorter operating times and potentially reduced blood loss. With our own data, biomechanical studies and clinical studies showing that the DHS/TSP has equivalent outcomes to the IMN, we suggest that both can be used in the management of unstable A3 intertrochanteric fractures, but we would suggest that the DHS/TSP may be an overall better choice due to its cost efficiency and shorter operating times.

### Limitations

Limitations of our study are centred on its retrospective design, leading to a lack of certain patient-reported outcome measures including patient mobility at discharge or calculation of objective hip performance scores such as the Harris Hip Score. This prevented full evaluation of patient outcomes, although RCTs have shown no significant differences between the two fixators [17]. We also do not have data on the fixation outcome, such as rates of non-union, as patients were not X-ray imaged post-operatively. However, our results still provide valuable insight into hospital resource usage and patient safety. Additionally, future studies could consider factors that impact time to fracture union and complication rates, such as engagement with physiotherapy and patient comorbidities.

One outcome that we could investigate in a prospective follow-up study would be the capability to perform activities of daily living, as this is rarely measured in these studies but is a very important outcome measure for patients, having a substantial impact on their quality of life. Along with patient pain, this can be measured at clinic follow-up appointments.

## Conclusion

Unstable intertrochanteric fractures are difficult to fix, with no clear recommendation on the choice of implant nationally. Overall, our data and the evidence from the literature show that patients with DHS/TSP perform just as well as those treated with an IMN for intertrochanteric fractures with insignificant differences in all of our post-operative measures. This supports national guidelines but crucially, our study identified significantly shorter operating times with the DHS/TSP. There was an increased requirement for blood transfusions in the IMN group when stratified to AO-OTA 31-A3 fractures, suggesting that the DHS/TSP may perform better in unstable fractures. There are therefore advantages to using the DHS/TSP across both types of fracture. With the DHS/TSP construct significantly cheaper than IMN, the former could become the widely adopted fixation method for unstable intertrochanteric fractures as well, becoming the recommended fixator for all intertrochanteric fractures. Further trials should be conducted to identify any additional differences between these two methods of fixation, to objectively identify the best fixator for A3 fractures. Ultimately, the results of this study discerns potential differences favouring the DHS/TSP over the IMN and further illustrates that the optimal fixator for intertrochanteric fractures is yet to be determined.
